# Targeting Epigenetic Modifications in Uveal Melanoma

**DOI:** 10.3390/ijms21155314

**Published:** 2020-07-27

**Authors:** Pooneh Chokhachi Baradaran, Zuzana Kozovska, Alena Furdova, Bozena Smolkova

**Affiliations:** 1 Department of Molecular Oncology, Cancer Research Institute, Biomedical Research Center of the Slovak Academy of Sciences, Dubravska Cesta 9, 845 05 Bratislava, Slovakia; pooneh.baradaran@savba.sk (P.C.B.); zuzana.kozovska@savba.sk (Z.K.); 2 Department of Genetics, Faculty of Natural Sciences, Comenius University in Bratislava, 841 04 Bratislava, Slovakia; 3 Department of Ophthalmology, Faculty of Medicine, Comenius University, 826 06 Bratislava, Slovakia; alikafurdova@gmail.com

**Keywords:** uveal melanoma, epigenetic therapy, DNA methylation, histone modifications, CRISPR-dCas9, epigenetic editing

## Abstract

Uveal melanoma (UM), the most common intraocular malignancy in adults, is a rare subset of melanoma. Despite effective primary therapy, around 50% of patients will develop the metastatic disease. Several clinical trials have been evaluated for patients with advanced UM, though outcomes remain dismal due to the lack of efficient therapies. Epigenetic dysregulation consisting of aberrant DNA methylation, histone modifications, and small non-coding RNA expression, silencing tumor suppressor genes, or activating oncogenes, have been shown to play a significant role in UM initiation and progression. Given that there is no evidence any approach improves results so far, adopting combination therapies, incorporating a new generation of epigenetic drugs targeting these alterations, may pave the way for novel promising therapeutic options. Furthermore, the fusion of effector enzymes with nuclease-deficient Cas9 (dCas9) in clustered regularly interspaced short palindromic repeats (CRISPR) associated protein 9 (Cas9) system equips a potent tool for locus-specific erasure or establishment of DNA methylation as well as histone modifications and, therefore, transcriptional regulation of specific genes. Both, CRISPR-dCas9 potential for driver epigenetic alterations discovery, and possibilities for their targeting in UM are highlighted in this review.

## 1. Biology and Molecular Subtypes of Uveal Melanoma

Uveal melanoma (UM) is the most common primary cancer of the eye, causing fatal liver metastasis in up to half of the patients [1,2]. The average annual incidence varies widely according to ethnicity or latitude between less than 1 to more than 9 per million population per year, with the highest incidence in white Caucasians [3,4]. Primary UM is treated with either surgery or radiation with a low local recurrence rate. However, there are no efficient therapies for metastatic UM, and as a result, most of the patients survive less than 12 months after metastases diagnosis [5]. A recent meta-analysis including 912 metastatic UM patients reported the median OS 10.2 months (95% CI 9.5–11.0) [6]. The UM tumors arise from melanocytes located in the uveal layer of the eye, with the choroid the most frequent site (82%), followed by the ciliary body (15%) and iris (3%) [7]. G protein subunit alpha q (*GNAQ*) and G protein subunit alpha 11 (*GNA11*) hotspot mutations, present in 83% of UM, are considered to be initiating events in UM tumorigenesis [8,9]. Recurrent cytogenetic abnormalities, including the most significant loss of one copy of chromosome 3 (M3) as well as 8p loss/8q gain, 6p gains, and 1p deletion, also hold prognostic potential [10]. Loss-of-function mutations in the *BRCA1* associated protein 1 (*BAP1*) gene, located on 3p21, accompanied by decreased *BAP1* mRNA and protein expression, have been identified in M3-UM, indicating that *BAP1* abnormalities are highly correlated with the development of UM metastases. Patients with *BAP1* mutations are generally younger, between 30 and 59 years, compared to the mean age at diagnosis, 62 years [11,12]. Gene expression profiling is considered an important prognostic tool that can predict metastatic risk with higher certainty than clinical stage or chromosome 3 status. A commercially available expression panel of 15 genes, established by Castle Biosciences, categorizes patients as Class 1 (low metastatic risk) or Class 2 (high metastatic risk) [13]. Class 1 patients are subdivided into Class 1a and Class 1b categories based on the key mutations and the number of 8q and 6p copies [2]. The 5-year screening of patients revealed significant differences in metastatic risk of Class 1a, 1b, and Class 2 UMs, which are 2%, 21%, and 72%, respectively [14]. Recently, Robertson and colleagues identified four molecularly distinct UM subtypes, two associated with poor-prognosis M3 and two associated with better-prognosis disomy 3 (D3). Two subgroups of M3 tumors possess diverse clinical outcomes and are related to different biological pathways. In the first one, DNA damage repair, hypoxia-inducible factor 1 alpha (HIF1A), and MYC signaling are more prominent, while high levels of mitogen-activated protein kinase (MAPK) and phosphatidylinositol 3-kinase (PI3K)/Akt are more noticeable in the other subgroup [15]. This highlights the necessity for tailored therapeutic strategies that target these subtype-specific molecular changes. Moreover, unsupervised consensus clustering of the most variable 1% of CpG probes brought in four methylation clusters strongly associated with prognostic groups. UM in DNA methylation clusters 2 and 3 were highly enriched (12 of 16 tumors) in *SF3B1*/*SRFR2* mutations, whereas *EIF1AX* mutant tumors were only present in DNA methylation cluster 1. Thus, D3 UM with *EIF1AX* versus *SF3B1*/*SRFR2* mutations conveys diverse DNA methylation patterns. M3 *BAP1*-aberrant UM tumors offered a single global DNA methylation profile [15].

## 2. Role of Epigenetic Changes in UM Progression

**Figure 1:**
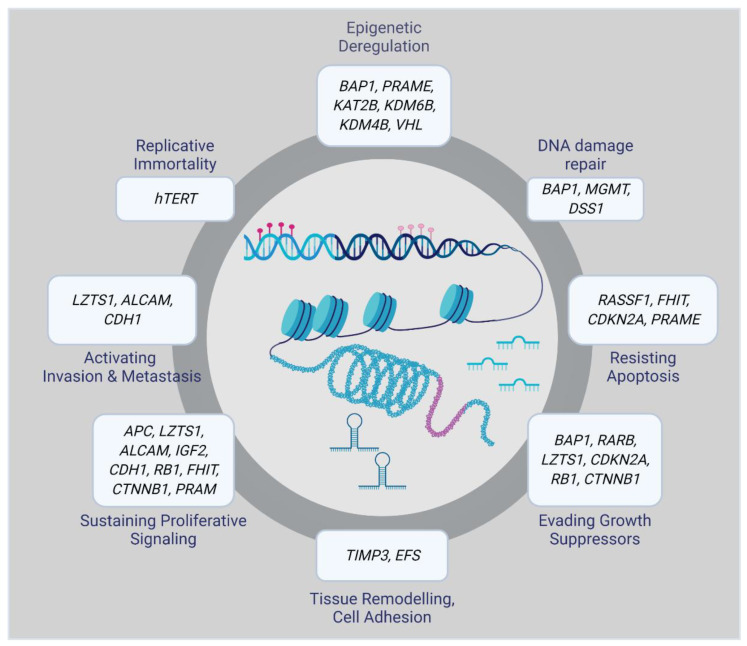
Epigenetically altered genes in uveal melanoma (UM).

Epigenetic alterations can result in aberrant gene regulation, thereby playing an essential role in tumorigenesis. Therefore, a complete overview of the epigenomic landscape is required to track the molecular events involved in the initiation and progression of a particular disease. Epigenetic changes that, for example, silence tumor suppressor genes or activate oncogenes include DNA methylation, histone modifications, and small non-coding RNAs. Many of them have been associated with UM initiation and progression (Figure 1) [16].

### 2.1. DNA Methylation

DNA methylation is a covalent modification with the addition of a methyl group [-CH3] to the cytosine residue in the CpG dinucleotide sequence. Methylation/demethylation is an essential mechanism in maintaining cell- or tissue-specific gene expression [17]. Correlation between gene expression profiles with global DNA methylome clusters identified so far in prognostically distinct UM tumors, suggests an epigenetic contribution to the underlying molecular pathology that produces this transcriptome [15,18].

Preferentially Expressed Antigen in Melanoma (*PRAME*) is an emerging epigenetic biomarker of metastasis in low-risk UM tumors [19]. It was shown that hypomethylation of specific CpG sites nearby the *PRAME* promoter resulted in its transcriptional activation, correlated with high metastatic risk in both classes 1 UMs [20]. There was a significant association between the high expression level of the Deleted Split hand/Split foot 1 (*DSS1*) gene and some clinical-pathological features. A recent study discovered that 64% of UMs showed higher expression of *DSS1* than healthy tissues. It was demonstrated that there is an inverse correlation between *DSS1* expression activity and the methylation status of its promoter [21].

Aberrant promoter hypermethylation of CpG islands plays a critical role in the inactivation of tumor suppressor genes in cancer [22]. Hypermethylation of *p16*, *TIMP3*, *RASSF1A*, *RASEF*, *hTERT,* and *EFS* genes have been reported in UM [23,24,25,26,27,28].

The Ras association domain family 1 isoform A (*RASSF1A*) gene is located on chromosome 3p21.3, and its absence or inactivation has been proved to be a contributing factor in UM tumor formation and progression [29]. It plays a crucial role in cell-cycle regulation, apoptosis, and microtubule stability [30]. Methylation of promoter sites of this gene control entry at the retinoblastoma checkpoint and inhibits cyclin D1 protein accumulation at the post-transcriptional level, leading to cell-cycle progression block from the G1 to the S phase. Though the methylation of *RASSF1A* may not be wholly responsible for UM development, it could be a contributing factor in UM tumorigenesis [29]. M3, which is related to the tumor’s metastatic capacity, has been reported in approximately half of all UMs. Considering the position of *RASSF1A* on the p21.3 region of chromosome 3, it could serve as a tumor suppressor gene whose silencing by methylation acts as a ‘second hit’ after monosomy occurs [26].

The Ras and EF-hand domain-containing (*RASEF)* gene, located on chromosome 9, region q21 is a candidate tumor suppressor gene. In 2007, UM cell lines and primary UM samples were screened for mutations in the *RASEF* gene region. The authors discovered that all cell lines and samples that did not express *RASEF* contained a methylated promoter, although those with *RASEF* expression lacked this methylation. They also demonstrated that methylation not only co-occur with low expression but also with a homozygous genotype. These findings propose that a combination of methylation and loss of heterozygosity may be the mechanism for loss of *RASEF* expression [31]. Homozygous tumors with a methylated *RASEF* promoter region tend to display reduced survival compared with heterozygous tumors without methylation, suggesting loss of heterozygosity might be related to the aggressive behavior of the tumor [31].

A study by Venza and colleagues in 2015 showed that DNA methyltransferase 1 (Dnmt1) and Dnmt3b have a preeminent role in *P16INK4A* (alias *CDKN2A*) repression. They demonstrated that epigenetic alterations in the *P16INK4A* and *P14ARF* (the alternative reading frame protein product of the *CDKN2A* locus) genes were frequently associated with cutaneous as well as UMs [32]. Moreover, it was demonstrated that *P16INK4A* is frequently inactivated by hypermethylation in both primary UM and UM cell lines, accompanied by a down-regulated expression of *P16INK4A* [23]. Both these studies also reported that loss of *P16INK4A* expression, attributable to CpG methylation, could be reversed when treated with the demethylating drug 5-aza-2′-deoxycytidine (decitabine). Interestingly, in UM patients who possess a tumor with a methylated *P16INK4A* promoter, metastasis tends to be more common. Therefore, modulation of abnormal methylation could be considered a valid target for UM treatment [23].

A recent study by Field and colleagues indicates that hypermethylation on chromosome 3 correlated with down-regulated gene expression at several loci, including 3p21 where *BAP1* is located. All Class 2 tumors contained a novel hypermethylated site within the *BAP1* locus, which reveals that *BAP1* itself is epigenetically regulated. In functional validation experiments, Bap1 knockdown in UM cell lines consisted of a similar methylomic repatterning with UM tumors, enhanced for genes involved in axon guidance, melanogenesis, and development [33]. This study provides evidence that *BAP1* loss leads to large-scale methylomic repatterning resulting in the Class 2 phenotype. Deciphering the role of epigenetic deregulation could explain the loss of melanocytic differentiation and gain of neural crest-like migratory behavior in Class 2 UMs.

### 2.2. Histone Modifications

Histone modification refers to the process of acetylation, phosphorylation, histone methylation, polyadenylation, ubiquitination, and ADP ribosylation, achieved by the relevant enzymatic activity [34]. Depletion of Bap1 protein trigger hyperubiquitination of H2A in melanoma cells and melanocytes, bringing about loss of differentiation along with the gain of stem-like properties [35,36]. On the contrary, treatment with histone deacetylase inhibitors (HDACis) in vivo in a xenograft model reversed the H2A hyperubiquitination, which may have therapeutic potential for inducing prolonged dormancy of micrometastatic UM disease [35]. While Hdac4 is localized to the nucleus in *BAP1*-mutant UM cells and the cells in which a *BAP1* mutation was introduced using CRISPR-Cas9, it is restricted mainly to the cytoplasm in *BAP1* wild-type UM cells and in normal human uveal melanocytes. Hence, Bap1 can inhibit the epigenetic function of Hdac4, at least in part, by diminishing its localization to the nucleus. Besides, short hairpin RNA (shRNA)-mediated depletion of Hdac4 in *BAP1*-mutant UM cells significantly impeded cell proliferation [37]. These findings suggest novel insights into the role of *BAP1* in development and cancer and propose HDACis as potential therapeutic agents for *BAP1*-mutant cancer’s treatment.

### 2.3. miRNA-Based Epigenetic Mechanism

Micro RNAs (miRNA) are among the most studied non-coding RNAs. They are short (17–22 nucleotides in length), phylogenetically preserved single-stranded RNA molecules involved in the gene expression regulation. Their dysregulation has been ascertained to confer resistance to apoptosis, promote cell-cycle progression, and enhance invasiveness and metastasis of many cancers [38]. A significant number of miRNAs have been shown to be differentially expressed in UM cell lines and tumor tissues [39]. The expression level of let-7b, miR-143, miR-193b, miR-199a, and miR-652 were proved to be increased in Class 2 UMs, so they can be used to differentiate between Class 1 and Class 2 UM tumors [40]. Radhakrishnan and colleagues identified distinct miRNAs present in metastatic UM and absent in non-metastasizing tumors [41].

Moreover, 96 miRNAs were reported altered in UM cell lines. Among them, 65 were downregulated, 28 upregulated, and 3 exhibited a different expression pattern [42]. The pleiotropic nature makes miRNAs particularly attractive drug targets. MiR-27a is an oncogenic miRNA overexpressed in various cancer types. Genistein was found to inhibit miR-27a expression in highly aggressive UM cells in vitro and in vivo, thus increasing the expression of its target gene *ZBTB10* significantly. Therefore, the authors hypothesized that genistein growth inhibitory activities can be mediated via miR-27a regulatory mechanism [43].

Treatment of UM cells with a DNA hypomethylating agent decitabine and HDACi trichostatin A (TSA), can regulate miR-124a expression level via epigenetic mechanisms [44]. Furthermore, decitabine was capable of enhancing miR-137 expression, which is generally epigenetically silenced during UM initiation [45], proving that individual epigenetic mechanisms can interact with each other. The list of UM-associated miRNAs (reviewed in [46,47]) is continually expanding along with the development of experimental methods and miRNA research tools. However, the identification of clinically relevant target genes and corresponding biological pathways will pave the way for their therapeutic targeting.

## 3. Current Therapeutic Approaches Available for UM

### 3.1. Primary UM Therapies

A historical approach to definitive, local treatment in primary UM is enucleation. This method is still appropriate for large tumors with extensive extraocular growth and a low probability of retaining vision. However, since in 2006, when the COMS study failed to prove a survival gain with enucleation compared to brachytherapy, there has generally been a movement toward vision- and eye-preserving modalities [48,49]. Tumor size and location, retinal detachment or invasion, and patient age and health status are the main factors affecting clinical management [2].

Two surgical approaches which offer better eye-preserving outcome are transretinal and transscleral endoresection, even though local recurrence rates are higher with transscleral resection than with brachytherapy or enucleation. Kivela and colleagues reported 6.1% of tumor recurrence after brachytherapy and 32.6% after transscleral resection similar to the other studies [50,51,52].

Radiation therapy, including brachytherapy, charged particle radiotherapy, and stereotactic radiotherapy, is the most commonly globe-preserving technique in the treatment of UM [2,53,54] Iodine-125 and ruthenium-106 are the most frequently used radioisotopes. Beside, Palladium 103 is rarely employed, and cobalt 60 (60Co) was used in the past [55,56]. However, brachytherapy could cause critical side effects, as well. Therefore, regular ophthalmologic follow-up must be carried out to monitor probable detrimental effects, including neovascular glaucoma, radiation-induced retinopathy, cataracts, and macular edema that can appear up to 5 years after therapy [56]. In 2014 Shah and colleagues showed that utilization of intravitreal anti-vascular endothelial growth factor (VEGF) after brachytherapy could weaken or delay the rate of moderate vision loss, macular edema, and poor visual acuity [57]. The use of charged-particle radiotherapy can improve local control, eye preservation, and disease-free survival in medium to large tumors or those in a location that may not be amenable to plaque brachytherapy [58]. Furthermore, a high rate of local tumor control (>95% at 15 years) [59] can be achieved without significantly worse complications than plaque brachytherapy.

Laser photocoagulation, photodynamic therapy, and transpupillary thermotherapy are laser methods that could be employed as primary therapy for small choroidal lesions [60]. However, their clinical utility is limited due to conflicting results and possible side effects [2].

Several clinical trials were performed for adjuvant UM settings, however, with no or marginal survival benefit. These include, for example sequential treatment with low-dose dacarbazine and interferon alfa-2b (NCT01100528) [61], fotemustine (NCT02843386) [62], adjuvant intra-arterial fotemustine [63], adjuvant interferon alfa-2a [64], or Bacillus Calmette–Guérin injections [65]. A recent phase I/II clinical trial of adjuvant ipilimumab in 10 high-risk UM patients showed that at 36 months follow-up, 80% of patients had no evidence of distant disease (NCT01585194) [66]. Adjuvant sunitinib treatment assessed retrospectively on the sample of 64 UM patients was associated with better overall survival (OS), especially in the group of patients under 60 years of age [67]. However, small sample size and retrospective design warrant further investigation of these results. Four adjuvant trials phase II/III are ongoing, focusing on combination immunotherapy (NCT02519322, NCT03528408), dendritic cells plus autologous tumor RNA (NCT01983748) and HDACi valproic acid (VPA) in comparison to sunitinib (NCT02068586).

### 3.2. Metastatic UM Therapies

Systemic chemotherapy, immunotherapy, liver-directed therapies, and targeted agents against the MAPK pathway are among treatments that have been evaluated in clinical trials for patients with advanced disease [2]. However, response rates are usually less than 10%, and no therapy has been confirmed to improve OS [68,69]. As a result, median progression-free survival (PFS) and OS for the metastatic patients are disappointingly low (3.29 months and 10.2 months, respectively) [53,70]. UM most commonly (93%) metastasizes to the liver [71]. Resection of hepatic lesions may offer long-term survival and cure in selected cases. Various other techniques e.g., radiofrequency ablation, stereotactic radiotherapy, regional chemotherapy such as hepatic intra-arterial infusion, and isolated hepatic perfusion or embolization are other liver-directed approaches that were tested with limited success [70]. Similarly, chemotherapy regimens such as dacarbazine, cisplatin, treosulfan, temozolomide, fotemustine, and various combinations, have been utilized in metastatic UM with poor outcomes. None of these agents has been shown to prolong survival response rates, which varied from 0% to 15% [72,73,74,75,76].

While progress in immunotherapy, especially immune checkpoint inhibitors targeting cytotoxic T-lymphocyte-associated antigen 4 (CTLA-4) and programmed cell death-1 (PD-1), have had a significant therapeutic benefit for metastatic cutaneous melanoma, it has not same clinical efficiency in metastatic UM [69]. The poor mutational burden observed in UM may, in some way, contribute to the limited success of the immune checkpoint blockade. Furthermore, the upregulation of immunosuppressive factors such as IDO1 and TIGIT might be partly responsible for treatment resistance suggesting a role for combination immune therapies [15]. Although retrospective analysis of 89 metastatic UM patients treated with ipilimumab plus nivolumab confirmed that dual checkpoint inhibition yields higher response rates than single-agent immunotherapy, 92% of patients discontinued treatment due to toxicity or progressive disease [77].

Targeted agents against the MAPK pathway is one of the therapeutic approaches in metastatic UM. *GNAQ/GNA11* mutations are fundamental for the activation of the RAS-ERK pathway. However, *BRAF* or *NRAS* mutations, which mediate sensitivity to the BRAF inhibitors vemurafenib and dabrafenib in cutaneous melanoma [78,79], are not common in UM [80]. The molecular profile of UM suggests treatments that target downstream components of the molecular pathways driving tumor growth, such as MEK and protein kinase C (PKC). Although there are no adequate results so far (response rates generally less than 10%). Two clinical trials are currently underway assessing the safety and anti-tumor efficacy of orally available PKC inhibitor LXS196 in patients with solid tumors harboring GNAQ/11 mutations (NCT03947385) and metastatic UM (NCT02601378). Preliminary results were published for 17 UM patients, of whom a partial response was achieved in two, stable disease (>6 months) in seven, while the progressive disease was detected in seven patients [81].

One of the highly selective potential inhibitors of MEK is selumetinib. Enhanced clinical outcomes with selumetinib compared to chemotherapy (temozolomide or dacarbazine) was demonstrated in a randomized, phase II study in 101 metastatic UM patients [82]. Patients who received selumetinib gained significantly longer PFS than those who received standard chemotherapy (15.9 vs. 7 weeks, *p* < 0.001). Subsequently, the phase III study enrolled UM patients who were categorized into two groups, selumetinib plus dacarbazine vs. dacarbazine alone. Unfortunately, this study did not reach its primary endpoint; there was no significant improvement in median PFS in the selumetinib plus dacarbazine than the dacarbazine alone (2.8 vs. 1.8 months, p = 0.32). Furthermore, objective response rate was not significantly increased (3.1 vs. 0%, *p* = 0.36). Efforts to optimize the effectiveness of MEK inhibition are continuing.

Some of the other agents targeting pathways, apart from MAPK, are gefitinib (an epidermal growth factor inhibitor) [83], lenalidomide [84], thalidomide (immunomodulator) [85], bevacizumab (VEGF-blocking antibody) plus interferon-α [86], bevacizumab plus temozolomide [87], aflibercept (a “decoy” receptor binding circulating VEGF) [88], imatinib (a KIT inhibitor) [89,90] carboplatin/paclitaxel/sorafenib (a multikinase inhibitor) [91] and sunitinib (a multiple receptor tyrosine kinase inhibitor) [92]. Nevertheless, none of these agents or combinations provided worthwhile outcome in metastatic UM.

## 4. Potential of Epigenetic Therapies in UM

Epigenetic processes, specifically DNA promoter methylation and histone modifications, are vital cellular events during tumorigenesis [93]. Though significantly explored in hematologic malignancies, in solid tumors, epigenetic therapeutic approaches remain in the rear [94]. Epigenetic dysregulation plays a crucial role in UM pathogenesis, by its genetic simplicity [18]. Recently it has been uncovered that a distinct global DNA methylation state is associated with the poor-prognosis subtype characterized by M3 and *BAP1* mutations [15]. HDACis induce morphologic and transcriptomic changes along with cell-cycle arrest, associated with lower metastatic risk in preclinical models [35]. Some epigenetic drugs have been used in preclinical studies (Table 1) and clinical trials (Table 2) for UM. Given the critical impact of epigenetic regulatory mechanisms in triggering metastasis, there is a rationale for adopting the epigenetic approach as a novel therapy for UM treatment that could potentially add to the recent progress in immune and targeted therapies.

### 4.1. Inhibitors of DNA Methylation

During DNA methylation, gene silencing occurs due to the adding methyl group to cytosine residue in CpG islands located principally in promoter regions. This phenomenon is regulated by the affected promoter. Aberrant gene silencing by DNA methylation has shown the ability to modulate cancer biology and cause drug resistance [104]. Decitabine is a powerful DNMT inhibitor (DNMTi) that appears to diminish the methylation process in numerous cancer cell lines (including melanoma), allowing the re-expression of genes that malignant cells are trying to turn off [105].

A recent examination investigated the safety of utilizing the demethylating agent decitabine by hepatic arterial infusion in patients with unresectable liver metastases. A study including 9 eligible patients, initiated treatment in a dose-escalation phase I clinical trial. The primary tumor types consist of four UMs, four colorectal carcinomas, one skin melanoma, and one epithelial ovarian cancer. Decitabine was applied at three different doses (two patients at dose 10, four at 15 and six at a 20 mg decitabine/m^2^/day). It was administered as a 1-h hepatic arterial infusion every 4 weeks for five successive days. Expression levels of 30 cancer test antigens (CTA) in pre-treatment and post-treatment biopsies from all cohorts were analyzed. The expression of 21 out of 30 CTAs after treatment escalated. Predominant treatment-related adverse events were grades 1 and 2 hematological toxicity. No patients experienced treatment-limiting detriment [98]. However, during study treatment or post-study exposure to immune checkpoint therapy, no objective tumor responses were observed in four patients with UM liver metastases.

### 4.2. Histone Deacetylase Inhibitors

Given that DNA is wrapped around histones, the acetylation of which mediates chromatin relaxation associated with a higher level of gene transcription, histone acetylation and deacetylation represent an essential part of the epigenetic regulatory mechanism. Hence, increased activity of HDAC, one of the main characteristics of oncogenic changes, can be potentially restored by HDACis. HDACis are compounds that were shown to induce growth arrest of transformed cells, cell death, angiogenesis inhibition, and terminal differentiation [106,107]. Human HDACs, grouped into four classes based on the similarity of DNA sequences and function include 18 different substances [108]. It has been proven that HDACis induce dormancy of micrometastatic disease through differentiation of UM cells and shift UM cells from Class 2 to the Class 1 signature [35]. Some HDACis such as VPA, TSA, tenovin-6, panobinostat, entinostat, depsipeptide, vorinostat, quisinostat, NaB, JSL-1, MC1568, and MC1575 have shown promising results in preclinical UM models [109].

VPA, which is now in the clinical trial [NCT02068586], is a well-characterized HDACi used for almost 40 years in epilepsy treatment and as an anti-cancer agent recently [110]. Gene expression profiling, allowing enrollment of high-risk Class 2 patients, about half of whom will develop overt metastatic disease within three years of eye tumor diagnosis, can be highly relevant for clinical testing [111]. The ability of VPA to reverse the H2A hyperubiquitination caused by Bap1 loss, and to shift the gene expression profile of Class 2 cells toward a Class 1 profile, inducing changes compatible with melanocytic differentiation suggests that Bap1 normally maintains melanocyte differentiation in UM cells and its function can be at least partially reversed in lack of Bap1 by increasing histone H3 acetylation [35].

Similarly, panobinostat has been demonstrated to induce morphological differentiation, G1 cell-cycle arrest, and a shift to a differentiated, melanocytic gene-expression profile in cultured UM cells. Not only does it inhibits the proliferation of UM cells, but also it significantly reduces the fraction of viable cells [35].

The effect of vorinostat or suberanilohydroxamic acid (SAHA) on acetylation of histone H3 and H4 to *P14ARF* and *P16INK4A* promoters in UM cells have been evaluated by Venza et al. SAHA treatment-induced H3 and H4 hyperacetylation at the *P14ARF* promoter, followed by increased *P14ARF* expression, caused significant reduction in UM cell growth, migration, and invasion [101]. Vorinostat was also tested in a phase II clinical trial for treating patients with metastatic or unresectable melanoma [NCT00121225]. Despite stable disease in a high proportion of patients and some early responses, the primary endpoint of response has not met yet [112].

TSA upregulates the expression level of miR-137, a tumor suppressor gene acting through the down-regulation of it targets *MITF* and *CDK6*. A similar effect was caused by the transient transfection of miR-124a, down-regulated in UM cells. The transfection inhibited cell growth, migration, and invasion, via repression of the potential targets of miR-124a *CDK4*, *CDK6*, *CCND2,* and *EZH2* in UM cells [44].

Depsipeptide is a very potent HDACi that inhibits cell growth, induces apoptosis, and declines the migration of viable UM cells in both primary and metastatic UM cell lines [95,96].

Tenovin-6 suppressed UM cells’ growth in vitro through the deacetylation of SIRT1 and SIRT2 in UM cells, thus activating p53 expression and inducing apoptosis. A combination of tenovin-6 with the conventional vinblastine, a chemotherapeutic agent used for systemic therapy of UM patients, inhibited the viability of UM cells. Furthermore, tenovin-6, decreased the level of intracellularly active β-catenin, blocks WNT/β-catenin signaling, and diminished the population of cancer stem cells [97].

In a recent research JSL-1, a novel HDACi was studied in vitro and in vivo. It effectively inhibited cell proliferation. JSL-1 increases the expression of pro-apoptotic BH3-only protein (BIM) in UM cells, bringing about the induction of apoptosis. JSL-1 suppressed migration and invasion of UM cells with MMP-2 reduction. JSL-1 also inhibited the growth of UM xenografts in NOD-SCID mice. Furthermore, it eliminated UM cancer stem-like cells, which are considered seeds of metastasis by blocking the canonical Wnt/β-catenin pathway, impairing self-renewal capacity, and declining percentage of ALDH+ cells [100].

### 4.3. Combination Therapy Using Epigenetic Drugs

Epigenetic drugs such as DNMTis, HDACis, histone methyltransferase inhibitors (HMTis) e.g., enhancer of Zeste homolog 2 inhibitors (EZH2is), and modifiers of miRNA expression such as antagomirs were shown to reduce the resistance of tumor cells to the natural killer and cytotoxic T cells, and enhancing the functions of antigen-presenting cells [113]. At present, increasing attention is paid to the newly combined therapeutic approaches employing epigenetic drugs or new molecular inhibitors and other therapies to promote the efficacy of cancer treatment [114].

The safety and tolerability combination of dual epigenetic therapy (DNMT and HDAC inhibition) with chemotherapy was explored in metastatic melanoma. In this trial, decitabine and panobinostat were combined with a known standard agent for metastatic melanoma temozolomide. 17 melanoma patients, including 11 cutaneous, 4 ocular, and 2 mucosal melanomas, underwent therapy with this combination. Despite limited efficacy, this trial was generally well-tolerated by the treated groups and appeared safe to be continued to Phase II. None of the patients experienced dose-limiting toxicity. A maximum tolerated dose was not reached, as well [115].

MEK inhibitors (MEKis) appear to have anti-proliferative activity in metastatic UM patients, though responses are short-lived. Gonçalves and colleagues recently evaluated a group of epigenetic inhibitors, including Disruptor of telomeric silencing 1-like inhibitors (DOT1Lis), EZH2is, lysine-specific demethylase 1 inhibitors (LSD1is), DNMTis, and histone acetyltransferase inhibitors (HATis) as a strategy to diminish escape from MEKi therapy in vitro [99]. The authors proved that decitabine dramatically enhanced the anti-proliferative activity of trametinib in cell viability, 3D organoid, and colony formation assays. A combination of two drugs MEKi-DNMTi principally affected the expression of genes involved in G1 and G2/2M checkpoints, cell survival, chromosome segregation, and the mitotic spindle formation. Induction of DNA repair or senescence does not differ from either drug alone. Instead, the expression level of the CDK inhibitor p21, and the BIM were increased. Likewise, the DNMTi-MEKi combination more efficiently suppressed the growth of MP41 cells in UM xenografts than monotherapy. Thus, DNMTi may improve the activity of MEKi in UM [99].

### 4.4. Third Generation of Epigenetic Drugs

So far, epigenetic drugs consisting of DNMTis, HDACis, HMTis, and modifiers of miRNA expression appear to be more efficient for hematological cancers rather than solid tumors, possibly since solid tumors originate from more-differentiated or even terminally differentiated cells with a reduced capability for epigenetic reprogramming [94]. Development of first-generation and second-generation epigenetic drugs, which are almost exclusively DNMTis or HDACis, following a ‘one size fits all’ approach together with the lack of predictive biomarkers for patient selection thereby, resulted in disappointingly low efficacy in patients with solid tumors. However, success with certain third-generation epigenetic drugs, used according to precision medicine paradigms, has provided new hope in epigenetic therapy. This group includes, among others, bromodomain and extra-terminal domain inhibitors (BETis), histone demethylase inhibitors (HDMis), HMTis, or protein arginine methyltransferase inhibitors (PRMTis) [114]. As they might have therapeutic potential in the treatment of UM, there is an increasing need to evaluate their efficiency. The first such attempt was made with BETi, which provides a novel therapeutic approach in UM treatment. The BET family of proteins, including BRD2, BRD3, BRD4, and BRDT bind to acetylated lysine residues on histone tails to direct the assembly of nuclear complexes regulate chromatin remodeling, DNA replication, and transcription [116,117]. More precisely, BRD4 can regulate oncogenic drivers such as *MYC* by binding to super-enhancers and non-coding DNA regions that are densely occupied by master transcription factors responsible for cell identity [102,118]. Since MYC amplification plays a crucial role in UM, it was hypothesized that BET inhibition therapeutic activity might be regulated via the down-regulation of MYC and MYC-dependent genes. Phase I and phase II clinical trial of BRD4 inhibitor (PLX2853) in various advanced malignancies, including UM is recruiting now [NCT03297424].

## 5. Epigenetic Editing

Due to the reversible nature of epigenetic changes, converting them to a “normal-like” chromatin landscape is feasible using “designed modular tools” [119]. The fundamental structure of editing tools includes a programmable DNA-binding domain and an epigenetic effector domain of interest. The zinc finger nucleases (ZFN), transcription activator-like effector (TALE), and nuclease-deficient Cas9 (dCas9) are three different programmable DNA-recognition domains which have been broadly used in epigenome editing, the most promising derivative technology of genome editing [120]. The epigenetic effectors are diverse, ranging from the writers, erasers, and readers, depositing, removing, or detecting DNA and histone modification marks as well as artificial transcription factors [121].

### Epigenetic Editing Using CRISPR/dCas9

The clustered regularly interspaced short palindromic repeats (CRISPR) bacteria primitively used associated protein 9 (Cas9) system for their defense against bacteriophages. Recently this system is receiving remarkable attention by its rising role in the treatment of genetic disorders and cancer [122]. CRISPR system consists of an RNA-guided Cas9 endonuclease protein, which has the potential to be repurposed for editing both the genome and epigenome with significant efficiency [123]. This system can dramatically accelerate cancer research progress, either by screening for novel therapeutic targets, developing cancer therapies or by functional genome/epigenome editing. Therefore, it can be considered a robust weapon in the arsenal of future cancer treatment [122,123].

Two de novo DNA methyltransferases (Dnmt3a/b) are responsible for establishing DNA methylation in mammalian cells and Dnmt1 for its maintaining. Furthermore, demethylation is feasible through oxidation of the methyl group by TET (ten–eleven translocation) dioxygenases to build 5-hydroxymethylcytosine, and then its recovery into unmodified cytosine by whether DNA glycosylase-initiated base excision repair or DNA replication-dependent dilution [124].

It was demonstrated that fusion of mutant form of Cas9 without endonuclease activity known as dCas9, with the Dnmt3a or Tet1 enzyme could provide a potent tool for targeted erasure or establishment of DNA methylation, respectively. In 2016 Rudolf Jaenisch group successfully repurposed the CRISPR/Cas9 system to rearrange the targeted genomic sequences’ methylation status. dCas9 protein was fused either to the Dnmt3a (dCas9-Dnmt3a) or the catalytic domain of Tet1 (dCas9-Tet1) to predictably edit the epigenetic state of specific sequences [125].

To methylate genomic sites of interest by raising the local Dnmt3a concentration dCas9 protein was fused to repetitive peptide epitopes (SunTag), recruiting multiple copies of antibody-fused Dnmt3a (dCas9-SunTag-Dnmt3a). The authors reported that dCas9-SunTag-Dnmt3a can significantly boost CpG methylation at the *HOXA5* locus in human embryonic kidney (HEK293T) cells and thus inhibit *HOXA5* gene expression, not to mention its minimal impact on the global DNA methylome and transcriptome [126].

**Figure 2:**
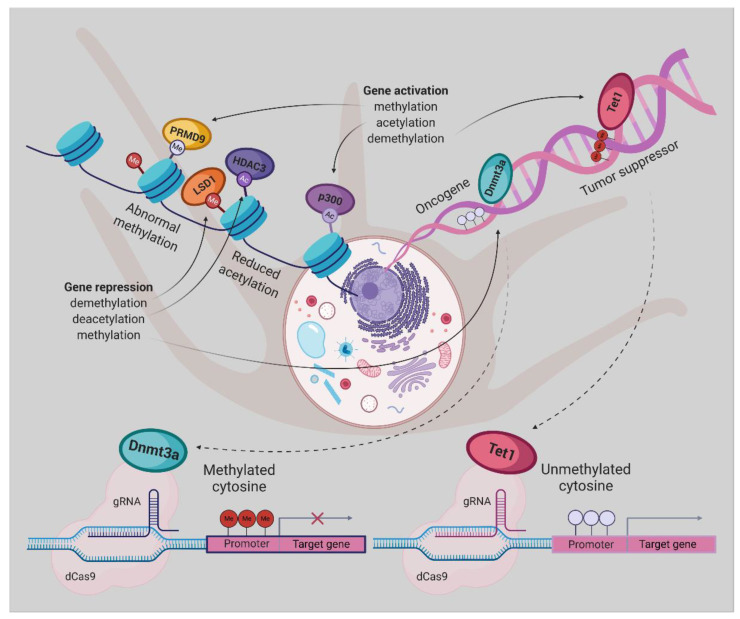
Epigenetic editing by CRISPR/dCas9, allowing for locus-specific control of epigenetically regulated gene expression, provides a more specific alternative to epigenetic drugs. DNA methylation or histone modifications can be restored using dCas9 protein fused or non-covalently bound to epigenetic effectors, derived from writers or erasers. Gene expression has been activated by DNA demethylation using Tet1, H3K27 acetylation by p300, or H3K4 trimethylation by PRDM9. The antagonistic effect can be achieved either by promoter methylation employing Dnmt3a, removal of a methyl group from H3K4me1/2 and H3K9me2 by LSD1 or deacetylation of H3K27ac by HDAC3 [127].

By gaining insight into the crucial role of molecular abnormalities, including epigenetic changes in cancer initiation, progression, and metastasis, there is a great demand for modern technologies to restore these aberrations. Therefore, in the coming years, the CRISPR-Cas system will play a notable role in cancer research. Repurposing this system to modulate the epigenome of cancer cells grant novel cancer therapies, albeit the employment of this platform, poses enormous challenges [123]. Taking together, dCas9-Dnmt3a/Tet1 could be an appropriate tool for implementing the new therapeutic strategies for UM (Figure 2) and other cancer types.

## 6. Conclusions

The overall mortality rate of UM remains high, regardless of progress in diagnosis and the therapy of the primary tumor. Thus, there is an extreme necessity for further research into alternative and modern forms of prevention and treatment. Epigenetics and epigenomics are new emerging research fields that start to unfold an era of contemporary approaches to improve clinical treatment and decline metastasis risk in UM patients. Although epigenetic therapies have been evolved to treat many human diseases, including cancer for more than 30 years, they more notably have relied on drugs that ubiquitously altered epigenetic marks. Notwithstanding, these epigenetic drugs could have severe side effects, since off-target genes may be affected. Moreover, clinical studies reported their disappointing efficacy in UM patients, so far.

Therefore, the establishment of new methods for restoring epigenetic modifications associated with high-risk UM is to be noted. Their success will be determined by the biomarker-directed approach, combining a new generation of epigenetic drugs with traditional and new therapies and the implementation of personalized strategies in clinical trials. Moreover, modern technologies such as dCas9-Dnmt3a/Tet1 system have the potential to start a new era in the treatment of UM and other cancers.

## Figures and Tables

**Table 1:** Preclinical studies focused on epigenetic drug anti-cancer effects in uveal melanoma.

Drug name	Function	Preclinical Model	References
Valproic acid	↑ Proliferation ↓ G1 cell cycle arrest ↓ Clonogenicity Shift Class 2 gene expression profile → Class 1	Primary UM cells, 92.1, OCM1A, Mel202, NOD SCID gamma mice	[35]
Trichostatin A	↓ Cell growth ↓ Migration and invasion ↓ Proliferation ↓ G1 cell cycle arrest	M619, C918, OCM-1, MUM-2b, -2c	[35,95]
Depsipeptide	↓ Cell growth ↑ Apoptosis ↓ Migration	M619, C918, OCM-1, MUM-2b, -2c	[95,96]
Tenovin-6	↓ Growth of UM cells ↑ *P53* expression ↑ Apoptosis ↓ Viability of UM cells ↓ β-catenin ↓ Cancer stem cells Blocks WNT/β-catenin signaling	92.1, Mel 270, Omm 1, Omm 2.3	[97]
Panobinostat	↑ Morphological differentiation G1 cell-cycle arrest ↑ Melanocytic gene-expression ↓ Fraction of viable UM cells Shift to a more differentiated phenotype	92-1, OCM1A, Mel202, NOD SCID gamma mice	[35]
Decitabine	↑ Anti-proliferative activity of trametinib ↑ Cell survival ↑ CDK inhibitor expression ↑ Pro-apoptotic BCL-2-like protein 11 (BIM) ↓ Growth of UM cell in xenografts ↑ Activity of MEKi	92-1, Mel270, MP41, Mel202, Mel290, CBySmn.CB17-Prdkc scid/j mice, Phase I clinical study	[98,99]
JSL-1	↓ Migration and invasion ↑ Expression of pro-apoptotic *BH3* gene ↓ Growth of UM xenografts ↓ Cell proliferation	92-1, Mel270, OMM1, OMM2.3, NOD-SCID mice	[100]
Vorinostat	↑ *P14ARF* expression ↓ UM cell growth ↓ Migration and invasion Shift Class 2 gene expression profile → Class 1	OCM-1, OCM3, 92-1, OMM-2.5, UMel-1, UMel-2	[101]
BRD4 inhibitors	↓ MYC and MYC-dependent genes ↓ Tumor growth ↑ Apoptosis	OMM1.3, Mel270, Mel202, SCID-beige and Vk*myc mice	[102,103]

**Table 2:** Clinical trials of epigenetic drugs in solid tumors, including uveal melanoma.

Drug Name	Recruitment Status	Phase	Dose	Regimen	Estimated Enrollment	Cancer Type	Clinical Trial Identifier
Vorinostat	Withdrawn	I	400 mg	Once a day for 15 days	10	UM	NCT03022565
Vorinostat	Suspended	II		Twice a day 3 days weekly for 4 weeks	40	UM	NCT01587352
Vorinostat	Completed	II		Once a day for 4 weeks	32	UM	NCT00121225
Entinostat	Active, not recruiting	II	5 mg	Once a day for a maximum of 24 weeks	29	UM	NCT02697630
Entinostat	Completed	II		Once a day, repeat every 2 weeks/once a day, repeat every 6 weeks	75	Choroid melanoma ^$^	NCT00020579
Valproic Acid	Recruiting	II		Daily for 6 months	150	UM	NCT02068586
PLX2853	Recruiting	I/II	Dose Escalation/ Expansion		166	UM *	NCT03297424

^$^ Advanced solid tumors or lymphoma; * Advanced solid tumors e.g., small cell lung cancer, ovarian clear cell carcinoma, Non-Hodgkin lymphoma, diffuse large b cell lymphoma, follicular lymphoma.

## Abbreviations

ADP	*Adenosine diphosphate*
BAP1	BRCA1 associated protein 1
BET	Bromodomain and Extra-Terminal motif
BETi	Bromodomain and Extra-Terminal motif inhibitor
BRAF	B-Raf Proto-Oncogene, Serine/Threonine Kinase
BRD2,3,4	Bromodomain Containing 2,3,4
BRDT	Bromodomain Testis Associated
Cas9	(CRISPR) associated protein 9
CCND2	Cyclin D2
CDK4	Cyclin Dependent Kinase 4
CDK6	Cyclin Dependent Kinase 6
-CH3	Methyl group
CpG	Cytosines followed by guanine residues
COMS	Collaborative Ocular Melanoma *Study*
CRISPR	Clustered regularly interspaced short palindromic repeats
CTA	Cancer test antigens
CTLA-4	Cytotoxic T-lymphocyte-associated antigen 4
D3	Disomy 3
dCas9	Nuclease-deficient Cas9
DNA	Deoxyribonucleic acid
DNMT	DNA methyltransferase
DNMTi	DNMT inhibitor
DOT1Li	Histone H3K79 methyltransferase inhibitor
DSS1	Deleted Split hand/Split foot 1
EFS	Embryonal Fyn-Associated Substrate
EIF1AX	Eukaryotic Translation Initiation Factor 1A X-Linked
EHMT2	Histone-lysine N-methyltransferase known as G9A
EZH2	Enhancer of zeste homolog
GNAQ	G Protein Subunit Alpha Q
GNA11	G Protein Subunit Alpha 11
HAT	Histone acetyltransferase
HDAC	Histone deacetylase
HDACi	Histone deacetylase inhibitor
HDM	Histone demethylase
HDMi	Histone demethylase inhibitor
HIF1A	Hypoxia-inducible factor 1 alpha
HMT	Histone methyltransferase
HOXA5	Homeobox A5
H2A	Histone H2A
H3	Histone 3
H4	Histone 4
IDO1	Indoleamine 2,3-Dioxygenase 1
KDM1Ai	Lysine-specific histone demethylase 1A inhibitor
LSDi	Lysine-specific demethylase 1 inhibitor
MAPK	Mitogen-activated protein kinase
M3	Monosomy 3
Me	Methylation
MEK	Mitogen-activated protein kinase kinase
MITF	Melanocyte Inducing Transcription Factor
miRNA	MicroRNA
MYC	MYC Proto-Oncogene, BHLH Transcription Factor
NRAS	NRAS Proto-Oncogene, GTPase
OS	Overall survival
PD-1	Programmed cell death-1
PFS	Progression-free survival
PI3K	Phosphatidylinositol 3-kinase
PKC	Protein kinase C
PRMT	Protein arginine methyltransferase
PRMTi	Protein arginine methyltransferase inhibitor
PRAME	Preferentially expressed antigen in melanoma
p16, P16INK4A	Cyclin Dependent Kinase Inhibitor 2A
P14ARF	The alternate reading frame protein product of the CDKN2A locus
RASEF	RAS and EF-hand domain containing
RASSF1A	Ras association domain family member 1
RNA	Ribonucleic acid
SAHA	Suberanilohydroxamic acid
SF3B1	Splicing Factor 3b Subunit 1
shRNA	Short hairpin RNA
TALE	Transcription activator-like effector
TET	Ten-eleven translocation protein
TIGIT	T-cell immunoreceptor with Ig and ITIM domains
TSA	Trichostatin A
TIMP3	TIMP metallopeptidase inhibitor 3
UM	Uveal melanoma
VEGF	Vascular endothelial growth factor
VPA	Valproic acid
ZBTB10	Zinc finger and BTB domain containing 10
ZFN	Zinc finger nuclease
